# Portable automated rapid testing for auditory assessment: repeated at-home testing in older adults

**DOI:** 10.3389/fdgth.2026.1686746

**Published:** 2026-05-15

**Authors:** Quentin Coppola, Lakshmi Kannan, Esteban Sebastian Lelo de Larrea-Mancera, Audrey A. Carrillo, Frederick Gallun, Susanne M. Jaeggi, Aaron R. Seitz

**Affiliations:** 1 Department of Psychology, Institute for Cognitive and Brain Health, Northeastern University, Boston, MA, United States; 2 School of Medicine, Oregon Health and Science University, Portland, OR, United States; 3 Brain Game Center for Mental Fitness and Well-Being, Northeastern University, Boston, MA, United States

**Keywords:** auditory assessment, central auditory processing, comfortability, older adults, peripheral auditory processing, remote assessment, tablet-based application, validation

## Abstract

**Introduction:**

Hearing challenges are prevalent in older adults and are associated with age-related cognitive decline. However, measuring age-related changes in hearing faces critical barriers related to accessibility and scalability. High-fidelity tests of central auditory functions are often unavailable to the individuals for whom auditory monitoring is most critical, particularly older adults.

**Methods:**

This study evaluated the feasibility and reliability of remotely administered assessments of auditory processing, delivered via the Portable Automated Rapid Testing (PART) platform, to measure hearing at home in older adults. Sixty older adult participants completed the study remotely with equipment mailed to their homes. They completed tasks across two separate timepoints assessing peripheral hearing as well as central auditory functions consisting of frequency modulation detection, speech-in-noise perception and spatial release from masking.

**Results:**

Most tasks in the PART platform exhibited moderate-to-high test-retest reliability and a high degree of acceptability. Findings suggested that PART is a promising tool for accessible auditory assessment.

**Discussion:**

PART is a promising tool for accessible auditory assessment in older adults. Future research should explore how central auditory processes relate to aging and cognitive health.

## Introduction

Hearing loss is one of the most prevalent sensory deficits among older adults, affecting approximately two-thirds of individuals aged 70 years and older (1, 2). It is increasingly recognized as a significant modifiable risk factor of cognitive decline, with an estimated 30%–40% of older adults with hearing loss experiencing cognitive impairment (3, 4, 54). Previous studies have also identified dysfunction in central auditory processing—hearing processed by the central nervous system—as highly predictive of clinical cognitive decline, including Alzheimer's disease (5–7). Further, peripheral hearing loss may also lead to functional and structural changes in the brain, leading to compensatory cognitive resource allocation and resulting in functional difficulties in everyday life (8). Likewise, studies suggest that individuals with cognitive decline are more likely to experience sensory or perceptual deficits, including difficulties in auditory processing (9, 10). Unidentified hearing difficulties not only affect communication but can also interfere with activities of daily living, including maintaining social relationships, navigating the environment, and participating in meaningful activities (11). Increased cognitive load due to effortful listening and social isolation due to communication difficulties can further degrade the synergy between hearing and cognition. These interactions between hearing and cognition suggest the need for early and accurate assessment of hearing difficulties as a potentially modifiable risk factor for dementia (3). As such, there is a great need for reliable, accessible measures to better understand auditory–cognitive relationships in aging and to detect hearing degradation earlier to inform timely intervention.

Traditional methods of assessing hearing emphasize tests of peripheral function, such as audiometric testing, typically conducted in controlled environments with expensive equipment and trained personnel. Furthermore, clinical audiograms, though widely used, assess hearing thresholds under idealized conditions and may fail to capture real-world auditory challenges such as speech-in-noise perception, which may be a stronger predictor of cognitive function (12). While valuable, in-person assessments limit the capability for large-scale or community-based research studies due to lack of scalability, particularly in underserved populations who may need this healthcare most (14). Alternatively, self-reported screening surveys, such as the hearing handicap inventory for the elderly (HHIE), are cost-effective but subjective and prone to reporting biases (13). As such, there is a pressing need for innovative approaches that bridge the gap between comprehensive audiometric testing and accessible, scalable alternatives.

Digital platforms address key limitations of traditional hearing assessment methods by combining accessibility, scalability, and reliability (14–18). The ability of digital assessments platforms to collect data remotely, without direct supervision of a clinician/experimenter, reduces logistic and geographical barriers, potentially enabling participation from a broader demographic, including older adults with mobility challenges (19). Several digital remote hearing assessment tools have been reported in the literature, such as digits-in-noise app-based testing, the World Health Organization's hearWHO, and other research platforms [see (20) and (14)]. These tools mainly focus on screening whereas, in contrast, PART provides a broader battery that includes both peripheral and central auditory measures, with the primary utility in research contexts. Further, Almufarrij and colleagues have found that out of 187 remote hearing assessment tools, only 12% had any data on reliability. In an effort to contribute to reliable and validated remote hearing assessment, our lab developed the Portable Automated Rapid Testing (PART) platform, a mobile application that facilitates remote data collection without compromising on the validity of the tasks compared with in-lab testing (16, 18).

PART is easily and freely accessible via the Apple App Store and Google Play Store and is fully customizable with a series of validated and adaptable auditory and neuropsychological assessments (21). PART has also demonstrated test–retest reliability among older adults with different hearing difficulties, including self-reported hearing loss, across various listening environments (22, 23). In addition, PART-based auditory tasks have shown cross-cultural applicability in both Spanish- and English-speaking populations (17, 24, 25). Collectively, this evidence supports PART's reliability for assessing auditory processing across diverse populations and settings. As such, PART exemplifies the potential of digital tools to assess auditory and neuropsychological function while providing scalable and accessible evaluations, making it particularly suitable for large-scale, multisite trials (15, 26).

The current work builds on recent research demonstrating PART's ability to reliably measure auditory processing by examining the self-administration of PART in home environments by older adults. The objective of the current study was to extend the previous findings by examining the feasibility and reliability of an extended, fully remote, digital hearing assessment battery targeting healthy older adults in the United States. To do this, PART hearing assessments were collected from a cognitively healthy older adult sample to evaluate if (i) data were within acceptable ranges given past work with similar tasks in similar populations; (ii) data were reliable between proximate timepoints; and (iii) participants found the tests to be tolerable based on their subjective response. We discuss opportunities for platform improvement and next steps for broadening participation in remotely administered tests of central auditory processing to better understand relationships between hearing and cognitive decline.

## Methods

### Participants

Eighty-two individuals aged 50–85 years Southern California were screened by phone to determine eligibility for the study and provided consent as approved by the UC Riverside Institutional Review Board. Participants were recruited via online advertisements on ClinicalTrial.gov, university mailing lists including the UC Riverside Aging Database and the UC Irvine Cradle-to-Career Registry, and flyers posted in local community centers. Potential participants completed a Qualtrics survey to determine preliminary eligibility (i.e., confirmation of older adult status) and then received the phone screen. To be included in the study, participants had to be able to understand and speak English to follow study procedures, with no self-reported speech, hearing, psychological, or neurological impairments that would interfere with their ability to provide informed consent or participate in the study. Exclusion criteria included (1) a formal diagnosis of dementia or other neurological disease, including Mild Cognitive Impairment (MCI), as confirmed by a total score below 17 on the Montreal Cognitive Assessment—Blind telephone version (T-MoCA), (2) a physical disability that would impede training procedures, or (3) a mental illness requiring treatment, and/or (4) significant absences during the study timeline.

Of the 82 screened participants, 68 successfully completed the first timepoint, and 63 successfully completed both timepoint one (T1) and timepoint two (T2). Of these, three participants had 50% unusable data from both sessions, resulting in analyses of 60 participants. Visualizations of missing and removed data (e.g., administration issue, outlier removal) are provided in Supplementary Figure S2. The final analytical sample had a mean age of 70.4 years (SD = 6.6; range = 58–84), was highly educated [years of education mean (SD) = 16.6 (4.0), and was predominantly female (77%)]. The racial composition was 75% White, 15% Black or African American, 4% Asian, and 6% Other or undeclared. Further, 9% of our sample also identified as Hispanic or Latino.

### Procedures

Participants were shipped study equipment, including a calibrated Surface Go 2 or 3 tablet, Sennheiser HD 280 Pro headphones, and detailed written instructions on how to use the tablet. Participants completed demographic surveys through Qualtrics, as well as auditory and neuropsychological assessments across two timepoints administered approximately 1 month apart. Each timepoint distributed the assessments across four experimental sessions over a week for a total of eight sessions. T1 took place within 1–2 weeks following consent, while T2 repeated all measures approximately 30–45 days later. Participants had on average 43 days in between assessments with a standard deviation of 13 days. The first experimental session was conducted remotely via Zoom by a trained research administrator, who instructed the participants on tablet use and task procedures across both sessions. Data from all tasks were collected digitally through the PART (https://ucrbraingamecenter.github.io/PART_Utilities/). Participants were asked to complete testing in a quiet location free of distractions, using the provided headphones for the Zoom call. Participants received a $150 Amazon gift card for their participation.

### Tasks

#### Self-report

##### Subjective task comfort

Participants provided comfort levels after each task using a Qualtrics survey. In particular, after each task, participants were asked the following questions: “Did you find the past task tolerable?” “Were you comfortable during the last task?” Their responses were categorized into four groups: “Yes,” “Maybe,” “No,” and “No response” (with missing or blank responses coded as “No response”). A comfort index was computed for each task by taking the frequency of the “Yes” responses and adding half the frequency of “Maybe” responses (Comfort Index = Yes + 0.5 × Maybe). Tasks with higher indices were rated as more comfortable, whereas tasks with lower indices were considered less comfortable.

##### Hearing Handicap Inventory for Elderly Screening (HHIE-S)

This 10-item self-report questionnaire measures the social and emotional effects of hearing loss. Scores range from 0 to 40, with higher scores indicating greater perceived impairment or auditory dysfunction. Scores between 0 and 8 suggest no significant hearing handicap, 10–24 suggest a mild to moderate hearing handicap, and 26–40 suggest a severe hearing handicap (13).

#### Peripheral auditory processing tasks

##### Comfortable listening levels

**Figure 1:**
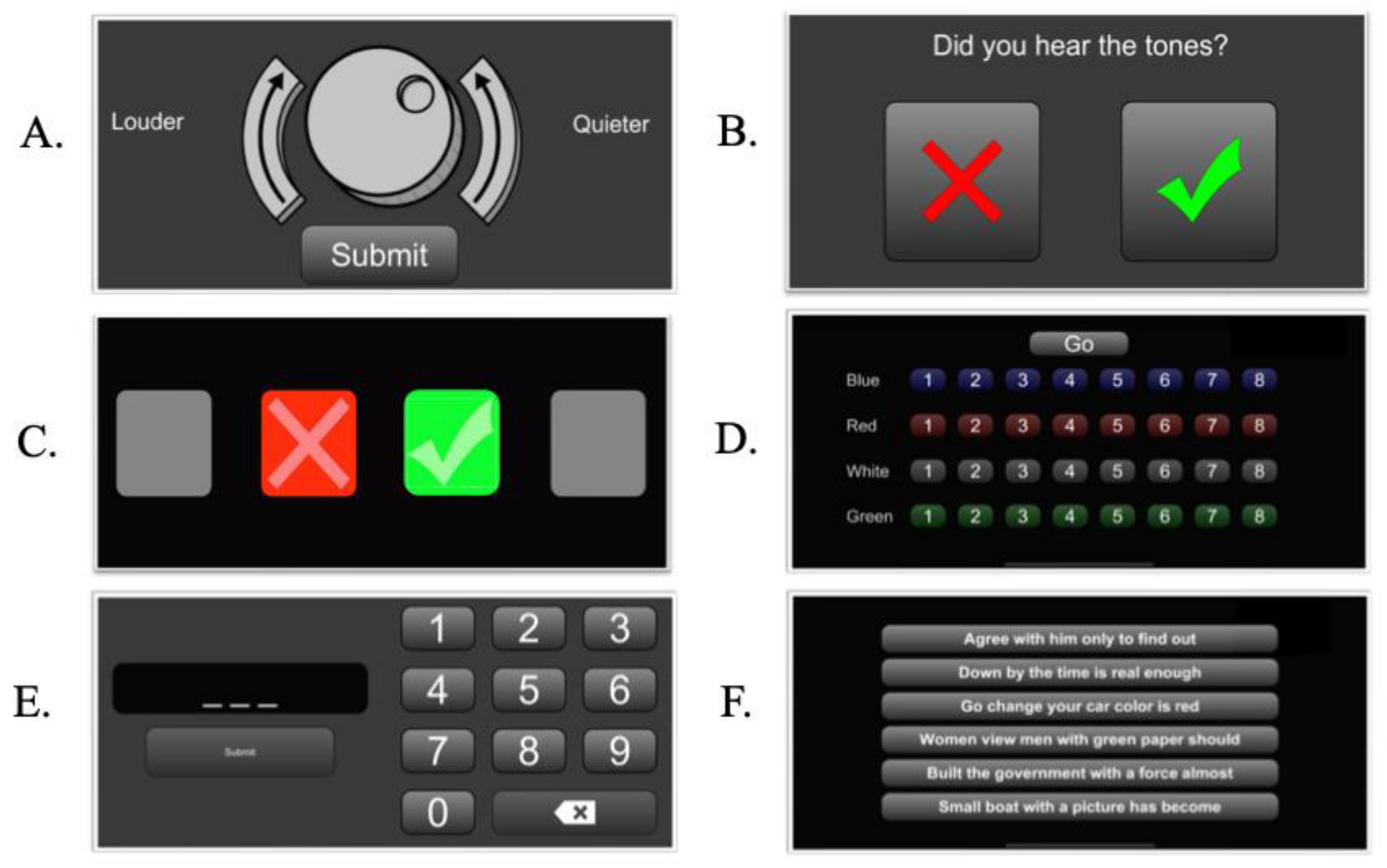
Participant-facing task response screens for auditory tasks in PART. **(A)** Comfortable listening levels task. **(B)** Pure-tone detection. **(C)** Four-interval, two-alternative forced-choice used for frequency modulation and spectrotemporal modulation. **(D)** Spatial release from masking. **(E)** Digits in noise. **(F)** Dichotic sentence identification.

A sound-level adjustment task measured comfortable levels of audibility of speech across various degrees of competition. In this task, participants were asked to adjust a dial to change noise volume to determine thresholds for comfortable listening and tolerable noise levels. The dial adjusted the background noise while participants listened to a target sentence presented at a constant level of 55 decibels sound pressure level (dB SPL) (Figure 1A). The target sentence was randomly drawn for each condition from the coordinate response measure corpus [CRM; (27)]. Noise consisted of multiple talker babble derived from the CRM, sounding like a cacophony of overlapping sentences with the structure “Ready  < call sign >  go to  < color >   < number >  now.” Participants were able to increase or decrease the volume of the babble by 1 dB, with the maximum level being 85 dB and minimum 0 dB. For the speech reception condition, which was labeled the “Masked” condition, participants were asked to increase the noise volume until the target sentence was too difficult to understand. In the “Comfortable” condition, participants were asked to adjust the noise to a level that was soft enough to understand the target sentence comfortably. Finally, in the “Uncomfortable” condition, which was repeated twice, participants adjusted noise to the maximum level they would be able to tolerate for an extended period while still understanding the target sentence. The thresholds for each condition were defined as the level of noise that the participant confirmed at the end of each condition, and subsequently, those values were recorded in dB SPL. The dependent variable used was the target-to-masker ratio (dB TMR), calculated by subtracting the sentence presentation level of 55 dB SPL from the final noise volume selected by the participant.

##### Pure tone detection

Participants' pure-tone discrimination thresholds were collected at 1,000 Hz for each ear individually, and at 500, 2,000, and 4,000 Hz diotically, with procedures modeled after clinical testing standards (28), although without clinically calibrated audiometers and sound treated environments. Participants were instructed to press a green checkmark if they heard a sound, or a red “x” if they did not (see Figure 1B). Stimuli were presented at an initial sound level of 50 dB SPL and then adapted using a modified Hughson-Westlake algorithm. If the first trial was incorrect, the tone level was increased by 20 dB SPL. Otherwise, the level was increased or decreased by 10 dB SPL until it reached 30 dB SPL, after which the step size changed to 5 dB, decreasing following a correct response and increasing following an incorrect response (29). Catch trials, where no sound would play, were added every five trials, and then every three trials, across each condition to evaluate engagement. If a participant had more than three false alarms in their catch trials, the task would terminate. The dependent variable was the estimated detection threshold at each frequency, determined as the last correctly detected level when either three consecutive “no” responses or 0 dB was reached.

#### Central auditory processing tasks

##### Frequency modulation

The frequency modulation (FM) tasks measured participants' sensitivity to tone frequency shifts under both binaural (dichotic) and monaural (diotic) conditions (30). A four-interval, two-alternative forced-choice task was used, in which a cue was presented in the first and last intervals, and either a cue or target was presented in the second or third interval (Figure 1C). For both FM conditions, a pure carrier tone was randomly selected between 460 and 550 Hz and was modulated sinusoidally at a rate of 2 Hz. The range of the frequency modulation, or modulation depth—also measured in Hz—was the adaptive parameter manipulated depending on participants' responses. FM depth had a base value of 20 Hz. In the diotic condition, the modulation phase was the same for both ears. In the dichotic FM condition, these waves were inverted between both ears, creating an interaural phase difference. This interaural phase difference, which is determined by FM depth, can be detected as a location change.

FM depth was manipulated using a two-stage 3-down/1-up staircase procedure that adapted exponentially, with a range between 0 and 10,000 Hz. The first stage (three reversals) employed step sizes (i.e., changes in FM depth) five times larger than those used in the second stage (six reversals), following procedures of Lelo de Larrea-Mancera et al. (15). The starting value for the task was 20 Hz, with an exponential step size, stepping down 2^1/2^ in the first stage and 2^1/10^ in the second stage. The dependent variable was the threshold level of the adaptive parameter, FM depth, representing the smallest amount of frequency variation that a participant can reliably detect approximately 79% of the time, measured in log_2_Hz [for more details, see (31, 32)].

##### Spectrotemporal modulation

The spectrotemporal modulation detection (STM) task measured participants' ability to detect sinusoidal amplitude modulations in broadband noise, applied to both the frequency spectrum at two cycles per octave and the time domain at 4 Hz (33, 34). STM also leveraged the same four-interval, two-alternative forced-choice paradigm as the FM tasks (Figure 1C). In this task, an unmodulated broadband noise with a spectral range of 0.4-8 Hz served as the carrier stimulus; participants were required to identify which of the four intervals contained the version of that noise that had been spectrotemporally modulated. The adaptive parameter—spectrotemporal modulation depth—was applied to the carrier tone using a logarithmic amplitude scale (measured in dB) and can be thought of as the degree to which simultaneous spectral and temporal cues manipulate the carrier tone (34, 35). The same two-stage adaptive paradigm described for the FM task was used, stopping after six reversals in stage two. The starting depth was 10 dB, which was stepped up or down by 2 dB. The dependent variable was the average STM depth where participants could reliably detect changes in both spectral and temporal dimensions, calculated by averaging the last six reversals. This threshold represents the minimum modulation depth required for participants to distinguish between the unmodulated carrier noise and the same noise modified in both its frequency content across different spectral bands and its amplitude fluctuations.

##### Spatial release from masking

Spatial release from masking (SRM) measured participants' ability to hear target speech during simultaneous competing speech (36, 37). In each condition of this task, a male talker produced a sentence containing a call sign to which participants were instructed to attend (e.g., Charlie), responding by tapping the correct row and column index denoted by color and number, respectively. An example stimulus sentence would be “Ready Charlie go to Blue Four now,” with “Charlie” being the call sign and “Blue Four” being the index (see Figure 1D).

The single-talker condition identified the participants' speech reception threshold (SRT), presenting only the target sentence without competing speech. This condition used a one-up, one-down staircase procedure with a 5 dB SPL step size. The base level of the target sentence was 55 dB SPL. The dependent variable—speech reception threshold (dB SPL)—was calculated by averaging all reversals (changes in difficulty direction) across all 20 trials.

Masking was introduced through two additional male talkers simultaneously speaking different sentences. Two masking conditions were tested: spatially colocated maskers (SRM_C_; all stimuli were presented at 0 degrees azimuth) and spatially separated maskers (SRM_S_; target presented at 0 degrees and maskers at ±45 degrees azimuth). Both masked conditions leveraged a linear progressive track, with a base threshold of 55 dB SPL, ending at 75 dB SPL, and increasing by steps of 2 dB every two trials. This provided two responses at each of 11 TMRs. The dependent variables for both colocated and separated SRM conditions were threshold TMRs, calculated by subtracting correct responses from 11 db. Positive TMR thresholds indicated that approximately 50% correct performance required maskers to be lower in level compared with the target, while negative thresholds indicated that approximately 50% correct performance was possible when the target was lower than the maskers. For a more detailed explanation of this procedure, see Gallun et al. (37) and Jakien et al. (38). SRM was calculated by subtracting the separated threshold from the colocated threshold, which served as the dependent variable. Positive values indicate improved speech-in-competition performance with spatial separation, while negative values indicate reduced performance with spatial separation.

##### Digits in noise

This task evaluated participants’ ability to identify triplet digits (0–9) generated as a sequence of random numbers with no filtering against a white noise background (39). Participants were instructed to enter the three numbers they heard into a number pad in the order they heard them (Figure 1E). Participants were allowed to correct any errant button presses by using a backspace button and then asked to confirm their response by pressing a button reading “Submit.”

This task used a one-up, one-down staircase procedure with an unequal up/down step sizes. Noise remained constant at 65 dB, while the base level of the target began at 65 dB SPL, ranging from 0 to 95 dB SPL across 25 trials. After an incorrect response, the target level increased by 5 dB, while a correct response decreased the target by 2 db. Thresholds (dB SPL) were calculated by taking the average of the target level at reversals across all trials and then subtracting by 65 dB (i.e., noise level), resulting in a TMR. Lower values indicated better speech-in-noise performance. Participants completed two runs of this task, and the average TMR across the two runs was used as the dependent variable.

##### Dichotic sentence identification

This task assessed dichotic hearing (40). Participants were asked to identify two sentences presented simultaneously at 70 dB (one in each ear) by matching them to a closed set of six possibilities displayed on screen (Figure 1F). Stimuli were randomly drawn from the synthetic sentence identification test, ensuring no repetition within pairs (41). All participants were given six trials, and if they did not achieve 100% accuracy on those trials, they completed six additional trials (6, 18). The dependent variable was accuracy across all trials.

### Statistics

For each task in the battery, we examined participants' subjective comfort levels, compared the current results to a comparable past sample, and assessed the task's test–retest reliability within the current sample. To assess tolerability, we calculated the percentage of participants who reported feeling comfortable by dividing the calculated comfort index by the total number of responses. Next, we graphically compared the distributions of each task from both timepoints in our current sample with those from comparative datasets that used similar procedures and samples, plotting the reported means plus or minus one standard deviation for each group alongside data from our current sample. We then examined test–retest reliability of our current sample by using complementary analyses that explored sources of random and systematic measurement variability. These consisted of (i) Pearson correlation coefficients to evaluate within-subject consistency (adjusting for session-related variance), (ii) repeated-measures *t*-tests to identify any systematic session effects, and (iii) Bland–Altman limits of agreement (LoA) to quantify absolute agreement and systematic bias (42, 43). Bias was calculated as the mean difference between the two timepoints, representing the systematic shifts, while LoA provided the range within which 95% of differences between repeated measurements were expected to fall. This multimethod approach for each task ensured a comprehensive evaluation of the reliability of the PART-based auditory battery.

## Results

Results are organized by self-report, peripheral auditory processing, and central auditory processing, as presented in the Methods section. Descriptive statistics and key statistics—including *t*-tests, correlations, bias, and LoA—are reported in Table 1. Scatter plots illustrating task performance across the two timepoints for each task are provided in Supplementary Figure S1.

**Table 1:** Summary statistics, paired sample *t*-tests, Pearson correlations, and limits of agreement analysis.

Task	*N*	T1_Mean (SD)_	T2_Mean (SD)_	*t* (*p*-value)	rho (*p*-value)	Bias	LoA
HHIE-S (Total)	59	12.4 (8.1)	11.5 (8.1)	2.83 (0.006)*	0.90 (<0.001)*	0.95	(−8.5, 10.4)
CLL_M_ (dB SPL)	59	68 (17.3)	66 (14.7)	0.63 (0.53)	0.30 (0.021)*	1.30	(−36.6, 39.2)
CLL_C_ (dB SPL)	60	46 (20.7)	47 (17.1)	−0.46 (0.65)	0.21 (0.107)	−1.42	(−48.4, 45.5)
CLL_U_ (dB SPL)	60	60 (12.4)	61 (13.1)	−0.49 (0.63)	0.23 (0.081)	−1.00	(−32.1, 30.1)
CLL_CU_ (dB SPL)	60	61 (13.9)	60 (14.7)	0.41 (0.680)	0.15 (0.25)	0.98	(−35.5, 37.5)
PT_Left 1kHz_ (dB HL)	57	36 (16.0)	30 (13.9)	3.53 (0.001)*	0.62 (<0.001)*	6.03	(−23.1, 35.2)
PT_Right 1kHz_ (dB HL)	56	37 (15.9)	30 (13.1)	3.03 (0.004)*	0.45 (0.001)*	6.90	(−28.6, 42.4)
PTdc_0.5 kHz_ (dB HL)	59	19 (14.5)	15 (10.4)	1.80 (0.076)	0.35 (0.006)*	3.75	(−27.1, 34.6)
PTdc_2 kHz_ (dB HL)	57	29 (16.9)	26 (14.3)	1.80 (0.072)	0.69 (<0.001)*	3.56	(−26.1, 33.2)
PTdc _4 kHz_ (dB HL)	59	26 (17.2)	32 (17.1)	1.74 (0.087)	0.60 (<0.001)*	3.00	(−30.5, 36.5)
FM_DC_ (log_2_Hz)	55	0.6 (1.8)	0.1 (1.4)	2.38 (0.021)*	0.55 (<0.001)*	0.54	(−3.4, 4.4)
FM_DT_ (log_2_Hz)	55	2.7 (1)	2.6 (0.7)	0.33 (0.740)	0.58 (<0.001)*	0.11	(−2.1, 2.3)
STM (dB SPL)	56	1.9 (0.8)	1.7 (0.6)	1.57 (0.120)	0.54 (<0.001)*	0.13	(−1.9, 2.1)
SRT (dB SPL)	59	46 (10.1)	47 (7.9)	2.29 (0.026)*	0.54 (<0.001)*	2.60	(−19.3, 24.5)
SRM_C_ (dB TMR)	59	3.8 (2.0)	3.8 (1.8)	0.18 (0.860)	0.36 (0.005)*	0.02	(−5.0, 5.0)
SRM_S_ (dB TMR)	60	2.5 (3.9)	1.4 (3.7)	2.33 (0.023)*	0.57 (<0.001)*	1.05	(−5.8, 7.9)
SRM (dB TMR)	59	−1.4 (3.2)	−2.4 (3.3)	−2.45 (0.017)*	0.50 (<0.001)*	0.97	(−7.4, 9.3)
DSI (accuracy)	58	80.3 (21.3)	83.6 (20)	−1.3 (0.120)	0.68 (<0.001)*	−3.33	(−60.7, 54.1)
DIN (dB TMR)	54	−19.0 (3.1)	−19.1 (3.6)	0.58 (0.583)	0.57 (<0.001)*	0.18	(−8.3, 8.9)

HHIE-S, hearing handicap inventory for elderly screening; CLL, comfortable listening levels task; M, masked; C, comfortable; U, uncomfortable; CU, confirm uncomfortable; PT, pure-tone threshold; DIN, digits in noise; DSI, dichotic sentence identification; SRT, speech reception threshold; SRM, spatial release from masking; C, colocated; S, separated; STM, spectrotemporal modulation; FM, frequency modulation; DC, dichotic; DT, diotic.

* Significance at an alpha level of 0.05.

### Self-report

#### Subjective task comfort

Comfort ratings were predominantly in the “comfortable” range across all tasks. Approximately 10–15 participants did not provide a response per task and were excluded from the analyses. These omissions were mainly due to administration errors in which the participant and test administrator skipped past the questions in the test battery. Participants generally rated the auditory tasks as tolerable, with the majority indicating “Yes” or “Maybe” when asked about task comfort. Most tasks demonstrated high tolerability, with dichotic sentence identification (DSI) rated most tolerable and SRM the least. Comfortability is presented where applicable for each task, with details of participants' comfort and tolerability levels presented in Supplementary Table S1.

#### HHIE-S

**Figure 2:**
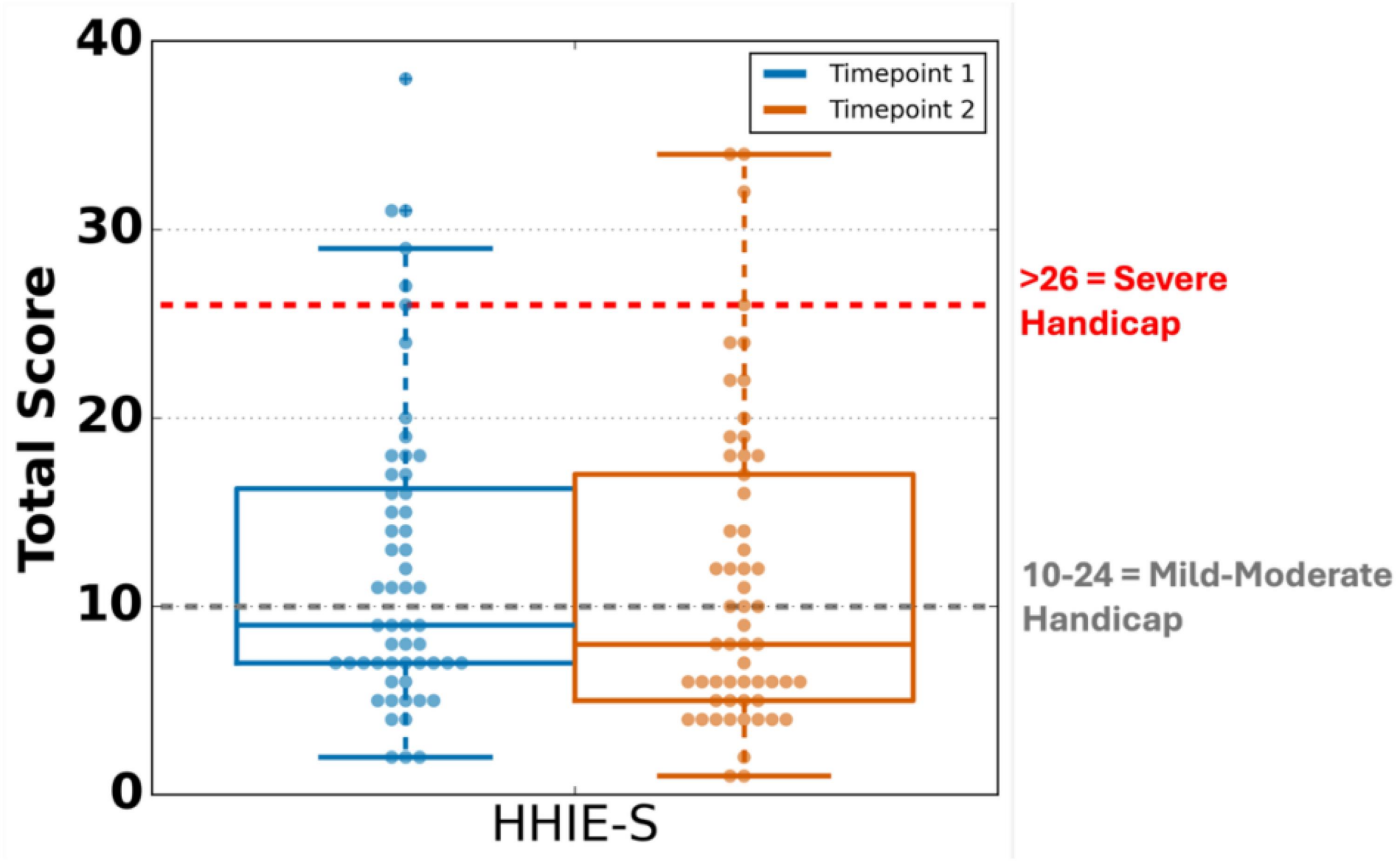
The Hearing Handicap Inventory for the Elderly Screening (HHIE-S): Distributions between timepoints. Scores of 0–8 suggest no significant hearing handicap, 10–24 suggest a mild to moderate hearing handicap, and 26–40 suggest a severe hearing handicap (13).

HHIE-S scores across both sessions indicated that 50% of participants scored 10 or higher, indicating mild to moderate self-reported hearing loss, while 10% scored above 26, indicating significant self-reported hearing loss (Figure 2). Notably, scores were highly correlated across timepoints, albeit with a slight bias toward T1, with participants reporting 0.97 points on average at T1 as compared with T2 [LoA: (−8.5, 10.4)]. These data suggest that we had mixed levels of hearing across the population; however, the bulk of our sample maintained mild to no hearing impairment.

### Peripheral auditory processing

#### Comfortable listening levels

**Figure 3:**
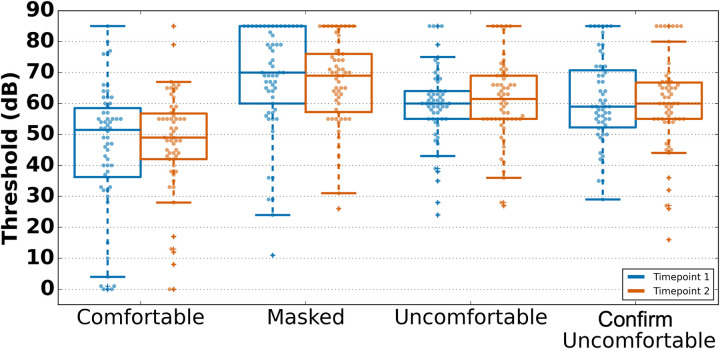
Hearing levels in the four comfortable listening level conditions across timepoints. Boxplot midlines show the median, boxes span the 25th to 75th percentiles, and vertical lines span the 10th to 90th percentiles.

The comfortable listening levels tasks were rated as comfortable by 86.7% of participants. Further ratings for each condition were consistent with instructed behavior, such that thresholds were higher for the masked and uncomfortable conditions when compared with the comfortable condition (Figure 3). However, LoA revealed within-subject variability across all conditions, most notably in the “Comfortable” condition. Despite this variability, all conditions saw small systematic bias (<1.5 dB) and non-significant *t*-tests across sessions. Moreover, correlations between timepoints were attenuated, with the only significant condition being the masked condition. Further, it is clear in the graphs that some participants adjusted outside of the expected range. In the future, it may make sense to add a floor to the test while also considering whether changes in instructions can reduce the subjectivity of ratings.

#### Pure-tone detection

**Figure 4:**
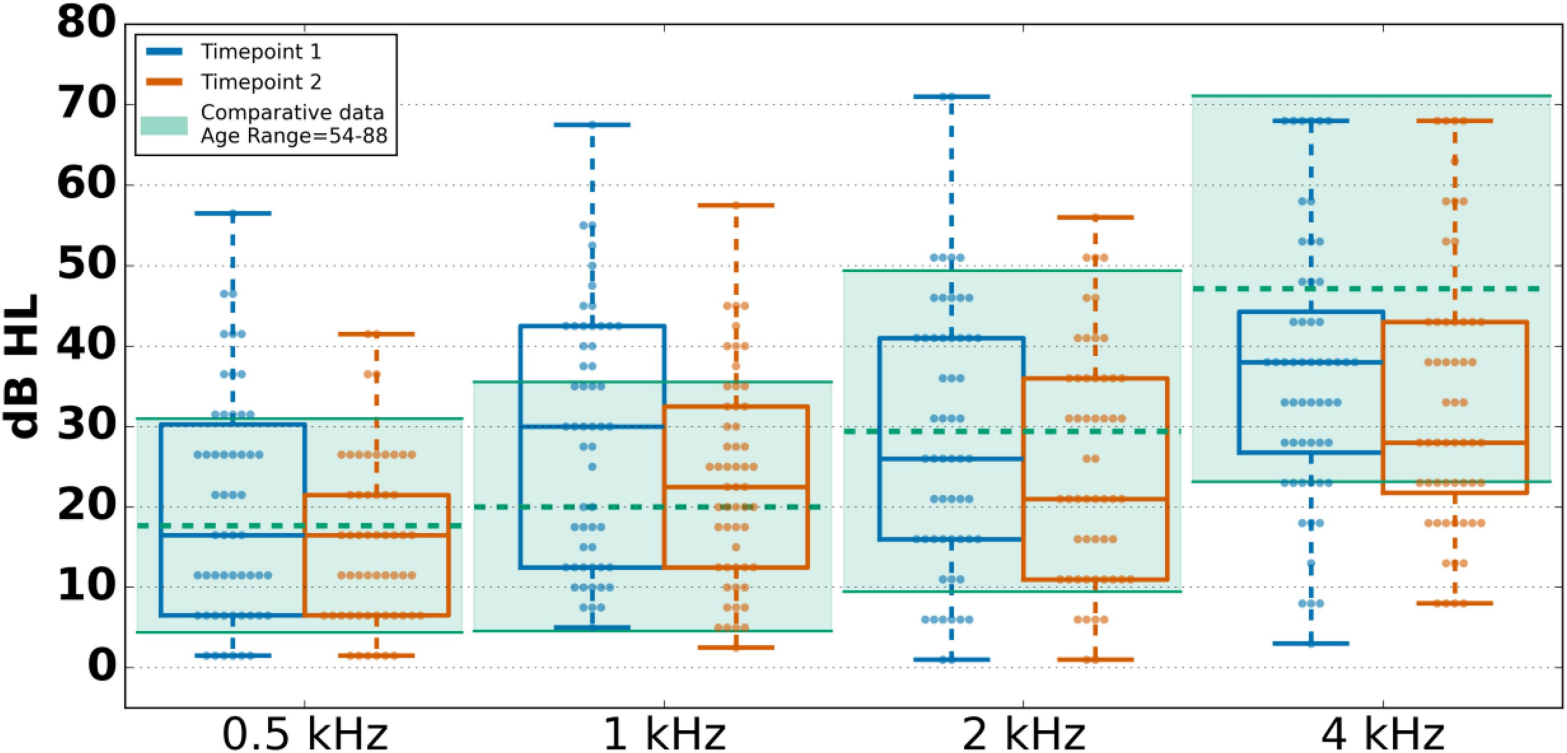
Diotic pure-tone thresholds across timepoints and comparative means ± one standard deviation. PT 1,000 Hz was calculated by averaging the detection threshold of both ears. Boxplot midlines show the median, boxes span the 25th to 75th percentiles, and vertical lines span the 10th to 90th percentiles. Horizontal dashed lines show the comparative mean and the highlighted regions show one standard deviation above and below that mean. Comparative data were collected from the longitudinal study of age-related hearing loss at the Medical University of South Carolina (44–46), comprising 1,704 participants aged 54–88.

Pure-tone detection was rated as tolerable by 95.3% of individuals who attempted the task. Although the PART-based pure-tone task is not a clinical measure, we compared current data to a large sample of older adults without self-reported hearing loss who underwent clinical audiometric exams (44–46). Our sample generally aligned with normative data from a large sample of older adults; however, our sample demonstrated notably lower thresholds on average for both timepoints, at 2 and 4 kHz (Figure 4). All conditions showed strong reliability with correlations ranging from 0.35 to 0.7 between sessions.

The second timepoint had lower variability and thresholds on average compared with T1, corroborated through the LoA analysis. This may demonstrate a familiarity effect where participants may have performed poorly in the first tests in the first session due to a lack of understanding of how to perform the task. Consistent with a familiarity effect, performance variability was greatest for conditions administered at the beginning of the session, specifically, both single-ear 1 kHz conditions [left: *t*(55) = 3.53, *p* = 0.001; right: *t*(56) = 3.03, *p* = 0.004] and the diotic 1 kHz condition averaged across both ears [*t*(54) = 2.39, *p* = 0.020], suggesting that participants may have been less familiar with task procedures during these early measurements. These data suggest that it would be important to have a familiarity phase for at-home testing in order to achieve more stable results.

### Central auditory processing

#### Frequency modulation and spectrotemporal modulation

**Figure 5:**
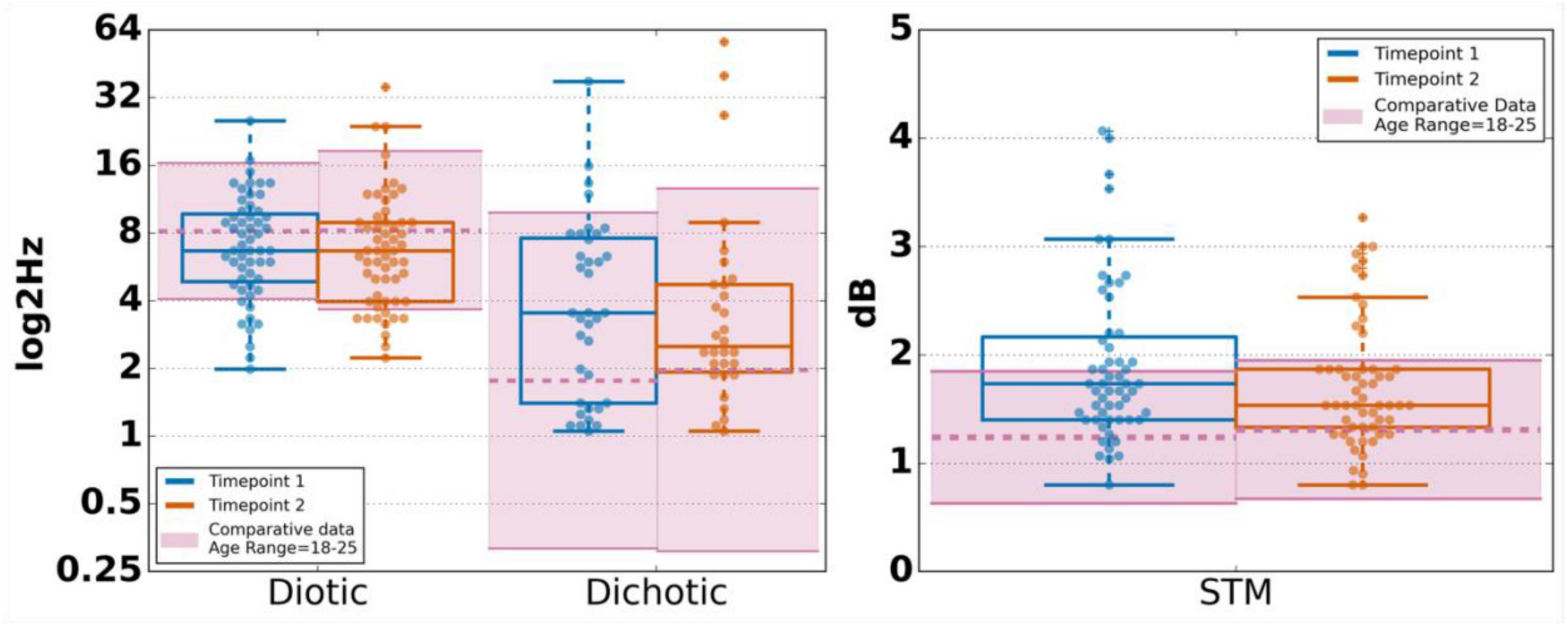
Frequency and spectrotemporal modulation across timepoints and comparative means ± one standard deviation. STM, spectrotemporal modulation; FM, frequency modulation. Boxplot midlines show the median, boxes span the 25th to 75th percentiles, and vertical lines span the 10th to 90th percentiles. Horizontal dashed lines show the comparative mean and the highlighted regions show one standard deviation above and below that mean. Comparative data are from Lelo de Larrea-Mancera et al. (16) age range = 18–25), collected across two sessions 1 week apart.

For each of the diotic FM, dichotic FM and STM tasks, approximately 97% of older adults in our sample found the tasks to be well tolerated. Correlations for both FM conditions were moderate, with 0.55 for the dichotic condition and 0.58 for the diotic condition. Given the lack of U.S.-based, age-appropriate norms for this task, the current sample (Figure 5) was compared with norms from PART tested in young adults (16). For the diotic FM task, older adult data generally matched that of younger adults; however, for the dichotic FM task, older adult data appeared worse than that of younger adults. For STM, although correlations were comparable to FM (rho = 0.54), young adult norms were significantly lower, pointing to a possible age effect [T1: *t*(84) = 5.88, *p* < .001. T2: *t*(104) = 4.56, *p* < .001]. For all three tasks, the limits of agreement analysis showed minimal bias (F_DC_ = 0.54; F_DT_ = 0.11; STM = 0.13) and small LoA [F_DC_ = (−3.4, 4.4); F_DT_ = (−2.1, 2.3); STM = (−1.9, 2.1)], comparable to prior in-lab and remote studies with younger adults (15, 16).

#### Spatial release from masking

**Figure 6:**
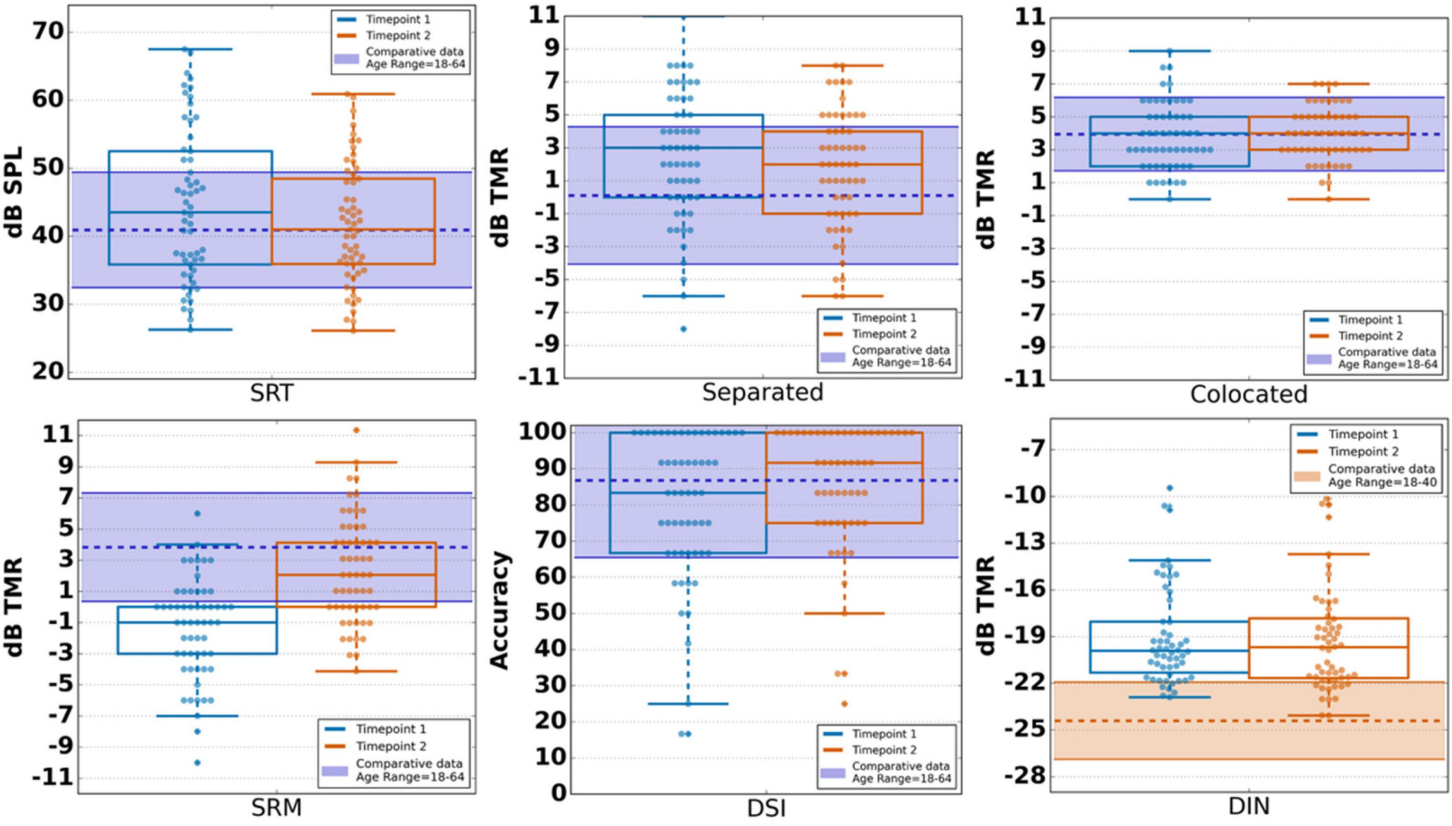
Spatial release from masking, dichotic sentence identification and digits in noise across timepoints and comparative means ± one standard deviation. Boxplot midlines show the median, boxes span the 25th to 75th percentiles, and vertical lines span the 10th to 90th percentiles. Horizontal dashed lines show the comparative means, and the highlighted regions show one standard deviation above and below those means. Comparative data for all spatial release from masking conditions (SRM; top row and bottom left panel), and dichotic sentence identification (DSI; bottom center) are from (18) (purple section: *N* = 89, age range = 18–64). Comparative data for digits in noise (DIN; center right) were collected from (25) (orange section: *N* = 56, age range = 18–40).

The SRM task was found to be comfortable by 79% of participants. SRM data were compared with recent work analyzing remote administration of an auditory PART battery across younger and middle-aged adults (18). Single-talker SRTs fell within similar ranges as the younger sample. In the SRM conditions, the current sample showed comparable thresholds in the colocated condition and higher thresholds in the separated condition relative to the comparative data (Figure 6, top row). Although correlations were significant for all SRM conditions across timepoints, the colocated condition was notably smaller (rho = 0.36) than the separated condition (rho = 0.57) and single-talker speech reception threshold (rho = 0.54). Further, t-tests revealed significant differences in the separated and spatial release conditions. LoA analysis corroborated this by revealing higher bias and greater variability in both the separated and spatial release conditions when compared with the colocated condition. Although our older adult sample demonstrated more variability, these effects follow a similar pattern demonstrated in younger adults using the SRM task in similar remote contexts (16).

#### Dichotic sentence identification

DSI was found to be highly tolerated (98.5%). Performance was compared with the same study used for SRM and generally fell within the same ranges, with many participants performing at ceiling (Figure 6, bottom center). DSI showed a significant correlation of 0.68 as well as a non-significant paired sample *t*-test. Minimal bias was demonstrated; however, the DSI demonstrated large LoA, with accuracy varying more than 50% on average across sessions. This effect may have been driven by outliers as well as individuals performing at ceiling across both timepoints.

#### Digits-in-noise

Digits in noise (DIN) was also found to be highly tolerated (100%); however, only seven participants responded to subjective comfort levels due to an administration error across subjects. DIN was compared with data recently collected remotely using PART with younger to middle-aged adults (25). Notably, the current sample demonstrates significantly higher TMRs across sessions compared with the younger sample, which may represent an age effect [T1: *t*(106) = 10.19, *p* < 0.001; T2: *t*(99) = 9.05, *p* < 0.001]. Otherwise, DIN showed good reliability through a significant correlation of 0.57, non-significant paired sample *t*-tests, and minimal bias with small LoA.

## Discussion

This study examined the acceptability and reliability of a self-administered, digital auditory battery in PART in a cohort of healthy older adults with typical hearing. Our results indicate that the auditory battery was well tolerated and exhibited good repeatability. Participants reported high comfort with the tasks; importantly, the distributions of scores in our current sample fell within the same broad ranges reported in comparable studies. Further, test–retest analyses showing our battery were consistent over a short period of time, aligning with previous work using the same tasks in a cohort of younger adults (15, 16, 25) and older adults (18, 24). However, although most tasks demonstrated moderate-to-high test–retest reliability, it was not uniform across the battery. Several tasks—including both left and right ear kHz pure-tone audiometry, dichotic frequency modulation, and some conditions of spatial release from masking—showed significant differences. Some tasks, most notably dichotic sentence identification, showed significant session differences and wide limits of agreement (see Table 1). Moreover, our comfortable listening levels task had also seen weak reliability across a short period of time. This could have been for a number of reasons, but this task was much more subjective in its nature compared with other tasks in our battery, which may lend itself to intraindividual variability (47). This weaker reliability shows that future work leveraging the comfortable listening levels task must analyze the degree to which these factors can be mitigated, or the degree to which this task may show utility within sessions rather than between sessions. With that said, previous work had not examined test–retest reliability for at-home testing in older adults, focusing instead on single-timepoint data collection or different populations. Our cohort was heterogeneous with respect to self-reported hearing handicap (HHIE-S mean = 10, SD = 8.1; see Table 1). Using conventional HHIE-S cutoffs, about half of participants scored greater than 10 (mild-to-moderate perceived handicap) and almost 10% scored 26 or more (severe perceived handicap). Although we refer to this sample as healthy older adults, we maintain a range of self-reported hearing difficulties, in line with aging literature. It has been demonstrated that older adults have a diverse set of perceived hearing handicap despite intact audiometry (5, 48). While these data support PART's suitability as an at-home assessment of auditory processing, there are several considerations that should be addressed to optimize its use.

A lack of understanding, or familiarity with these tasks, may have contributed to the poorer performance seen in some tasks. Performance tended to show bias toward the first timepoint, indicating generally worse performance. This may suggest that task familiarity likely plays an important role in task performance—specifically in the auditory measures—and that a combination of clearer tutorials, additional practice trials, and/or multiple sessions of testing may be required to achieve the most reliable performance. We note that while this issue of task familiarity may also play a role in assessments, an advantage of at-home assessment is that there are more opportunities for repeated testing to ensure that there are stable results. However, a target for future work will be to determine how better tutorials and practices can lead to more stable initial results and evaluate the degree to which practice effects may impact the interpretation and administration of these batteries. Importantly, an advantage of at-home testing is that participants can conduct tests multiple times in cases where there is a question of the reliability of an estimate.

Another issue that will be important to address is developing a greater set of normative data including populations with specific hearing challenges. The current study was designed to address initial acceptability of at-home testing of auditory processing, and neither our sample nor our comparative samples included participants who had existing diagnoses of a speech or hearing disorders. Further, we removed data points falling more than three standard deviations from the group mean. However, this limited our ability to address the extent to which the testing battery can reliably detect specific hearing deficits that may be found in the general population. Future work should investigate how these tasks perform in individuals with clinical or near-clinical central auditory dysfunction and the extent to which they are sensitive to detect clinically relevant changes in hearing when used longitudinally.

Moreover, our sample was predominantly White, educated, and female, which constrains generalizability. Prior work has demonstrated differences in manifestations of hearing loss across men and women (49), as well as disparities in interfacing comfortability with technology across varying socioeconomic status, (50) education (51), and race and ethnicity (51). Importantly, our comparison data were stratified similarly in terms of age and race, yet not in terms of socioeconomic status, biological sex, or education. Future efforts to use these tasks in PART should aim at including a larger population of individuals with lower education levels and minority backgrounds.

Relatedly, it will be important to determine effective use patterns for tools like PART in assessing people's hearing needs. A key advantage of PART is that it can easily be conducted at home and has been validated for use with participant-owned devices. This provides future opportunities to examine the utility of using PART both as a screener and as a monitoring tool. Further, PART is highly configurable, making it straightforward to re-administer specific tests of concern to determine whether poor performance reflects a genuine hearing deficit, or is attributable to task unfamiliarity, difficulty understanding instructions, or other technical factors that could affect task performance. Moreover, an advantage of these tasks is that—apart from the comfortable listening levels tasks—good task performance generally indicates good hearing ability. This is exactly what is desired for screening tasks where false positives—in case of poor performance—are more likely than false negatives—in case of good performance. PART can help flag cases of potential concern that can be followed up with clinically assisted assessment. With that said, the current work is only a preliminary step in the effort to validate PART across lifespan. Notably, this study does not validate remote testing against clinic-standard audiometry within the same participants; rather, the present study examined at-home repeatability. Recent work in adults (aged 25–60) with and without self-reported hearing difficulty found that data quality in online testing settings using PART, including individuals who self-report hearing dysfunction, is comparable with data from laboratory settings (52). Although promising, future studies should aim to validate this finding strictly in older adults using a within-subject, in-person versus remote design.

In addition, there are still aspects of scalability that need to be addressed for widespread use. In the current work, although participants were generally able to self-sufficiently administer the second timepoint of the battery, researchers helped with instructions for the first half. Further, we did run into non-trivial administration issues regarding data availability. Over 20 participants were either partially or fully excluded from the analysis due to incomplete data, either due to participant confusion during early sessions (immediately after onboarding) or researcher skip errors during supervised sessions. A total of 10 data points across all tasks of all participants (less than 1% of our data) suffered technical errors when downloading data from PART, resulting in exclusion. Future work will be required to establish the extent to which the entire battery could be self-administered, something that may possibly increase adherence, which is likely to be an easier target given the advance of easy-to-configure AI assistants.

While the current work focused primarily on peripheral and central auditory processing tasks, the PART platform also contains a rich collection of neuropsychological measures, as well as tests of vision, executive functions, cognitive control, and decision-making. This provides a unique opportunity to investigate relationships between central auditory processing and other cognitive functions. For example, recent work has demonstrated PART’s utility in evaluating executive functioning in Spanish-speaking older adults who have been diagnosed with Parkinson's disease, mild cognitive impairment, and dementia (10, 19). Future work aims to further elucidate the relationships between key auditory and cognitive processes, particularly in older adults where decline in both domains is a pressing concern.

In conclusion, as we move toward more accessible and scalable assessment methods, reliable hearing assessment tools such as PART show promise to improve precision measurement, which is crucial for understanding individual differences in both healthy and clinical populations. PART has the potential to serve as an accessible platform for assessing auditory function in older adults in a self-administered, home environment with affordable equipment. Most tasks demonstrated acceptable reliability between sessions in line with past work; however, more work needs to be done to establish the degree of familiarization necessary with older adult participants to ensure stable results with PART assessments. Further, more work is needed to establish validity of PART for screening and monitoring auditory function in clinical and subclinical populations. Future work should also elucidate how key considerations—such as individual differences in baseline central auditory processing—can affect the reliability of hearing tests administered in PART. The continuing validation of PART as a remote testing platform represents a significant contribution to the field of auditory assessment, particularly in the context of accessible, open-science tools for research. Older adults may be particularly well served by open-sourced remote testing technologies, as limitations with mobility and transportation may prevent many individuals from participating in lab settings (53).

## Data Availability

The raw data supporting the conclusions of this article will be made available by the authors, without undue reservation.
