# Correction: Portable automated rapid testing for auditory assessment: repeated at-home testing in older adults

**DOI:** 10.3389/fdgth.2026.1897310

**Published:** 2026-07-21

**Authors:** Quentin Coppola, Lakshmi Kannan, Esteban Sebastian Lelo de Larrea-Mancera, Audrey A. Carrillo, Frederick Gallun, Susanne M. Jaeggi, Aaron R. Seitz

**Affiliations:** 1 Department of Psychology, Institute for Cognitive and Brain Health, Northeastern University, Boston, MA, United States; 2 School of Medicine, Oregon Health and Science University, Portland, OR, United States; 3 Brain Game Center for Mental Fitness and Well-Being, Northeastern University, Boston, MA, United States

**Keywords:** auditory assessment, central auditory processing, comfortability, older adults, peripheral auditory processing, remote assessment, tablet-based application, validation

**Figure correction**

There was a mistake in figure 6 as published regarding the normative data from Lelo de Larrea-Mancera (2025a). The normative reference values for the colocated and separated conditions of spatial release from masking were inadvertently reversed in the figure. The corrected figure 6 appears below.

**Figure 6:**
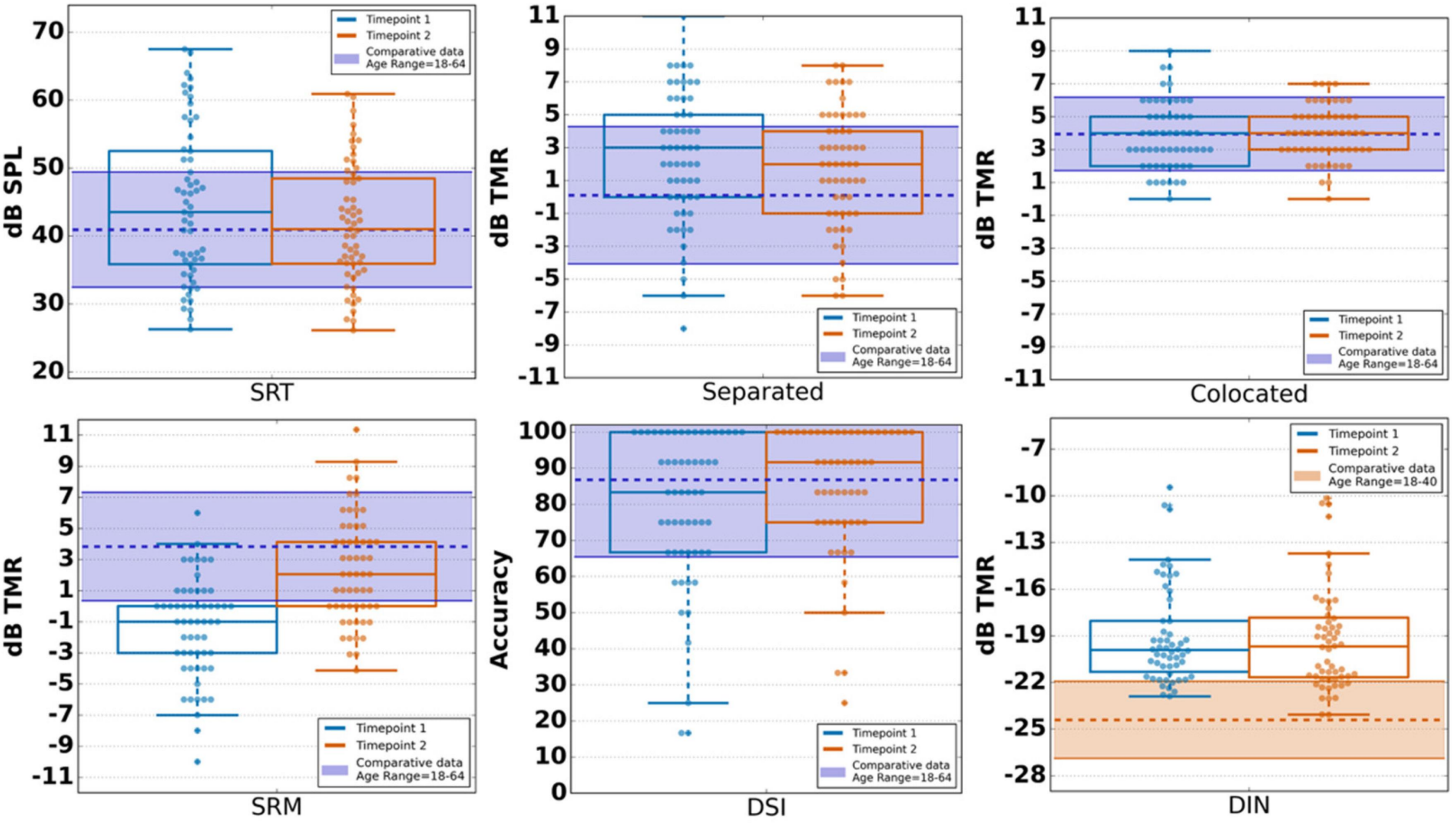
Spatial release from masking, dichotic sentence identification and digits in noise across timepoints and comparative means ± one standard deviation. Boxplot midlines show the median, boxes span the 25th to 75th percentiles, and vertical lines span the 10th to 90th percentiles. Horizontal dashed lines show the comparative means, and the highlighted regions show one standard deviation above and below those means. Comparative data for all spatial release from masking conditions (SRM; top row and bottom left panel), and dichotic sentence identification (DSI; bottom center) are from (18) (purple section: *N* = 89, age range = 18–64). Comparative data for digits in noise (DIN; center right) were collected from (25) (orange section: *N* = 56, age range = 18–40).

Due to the normative reference values being swapped, this changes the interpretation of the text in the Results. A correction has been made to the section **Results**, *Spatial release from masking*:

The SRM task was found to be comfortable by 79% of participants. SRM data were compared with recent work analyzing remote administration of an auditory PART battery across younger and middle-aged adults (18). Single-talker SRTs fell within similar ranges as the younger sample. In the SRM conditions, the current sample showed comparable thresholds in the colocated condition and higher thresholds in the separated condition relative to the comparative data (Figure 6, top row). Although correlations were significant for all SRM conditions across timepoints, the colocated condition was notably smaller (rho = 0.36) than the separated condition (rho = 0.57) and single-talker speech reception threshold (rho = 0.54). Further, t-tests revealed significant differences in the separated and spatial release conditions. LoA analysis corroborated this by revealing higher bias and greater variability in both the separated and spatial release conditions when compared with the colocated condition. Although our older adult sample demonstrated more variability, these effects follow a similar pattern demonstrated in younger adults using the SRM task in similar remote contexts (16).

