# Supplementary material for: Portable automated rapid testing for auditory assessment: repeated at-home testing in older adults
Source: Front Digit Health. 2026 May 15;8:1686746. doi: 10.3389/fdgth.2026.1686746 (PMC13219345; doi:10.3389/fdgth.2026.1686746)
Supplement: Supplementary file 1 [file Datasheet1.docx]

Supplementary Materials


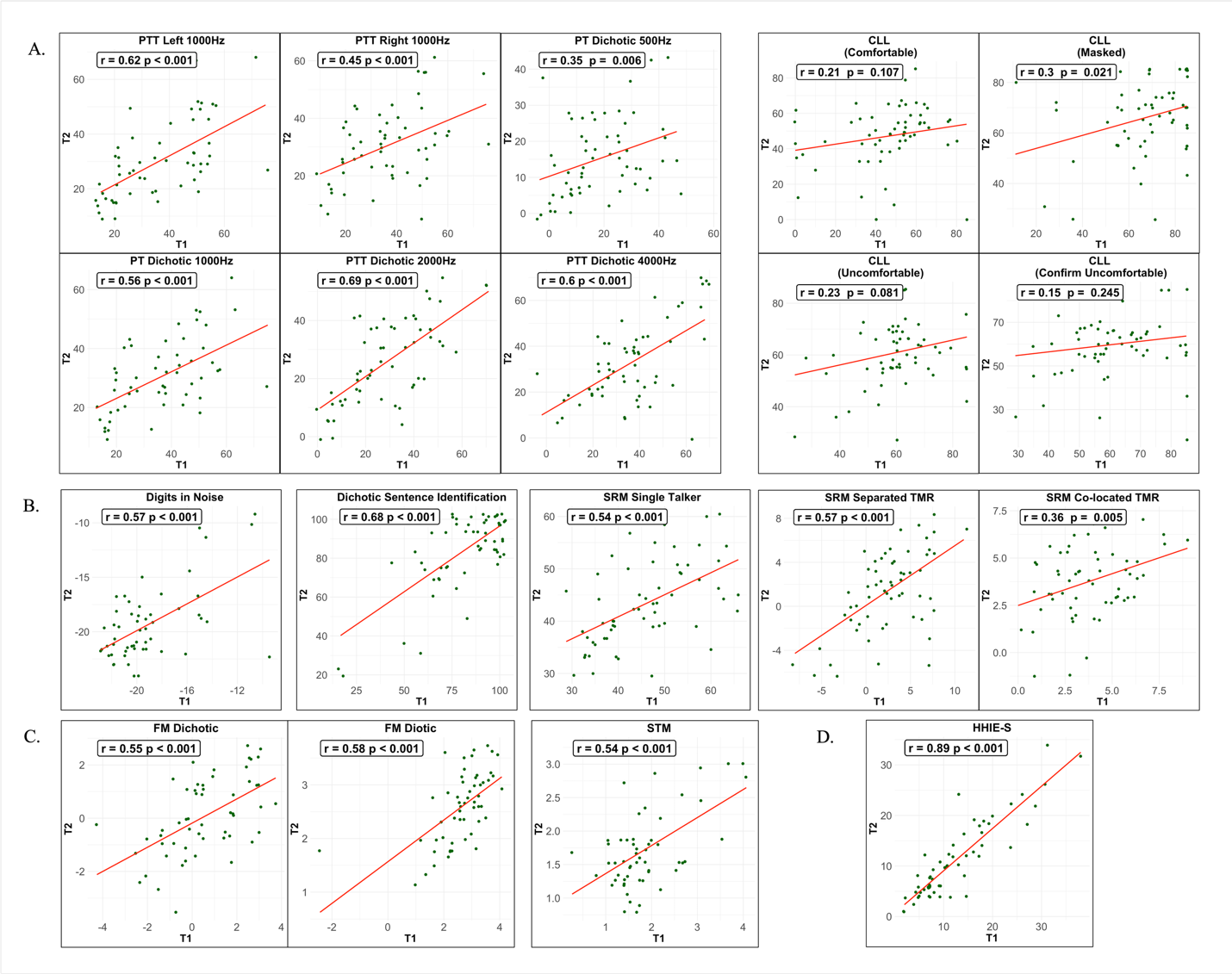


Figure S1. Scatterplots of All Tasks Compared Across Timepoints. A. Shows Audibility Tasks (Pure Tone Thresholding and Comfortable Listening Levels (CLL)). B. Speech-in-Competition Tasks (Spatial Release from Masking (SRM), Digits in Noise, Dichotic Sentence Identification) C. Spectrotemporal Fine Sensitivity (Frequency Modulation/FM and Spectrotemporal Modulation/STM) D. Self-Report (Hearing Handicap Inventory for the Elderly (HHIE-S))


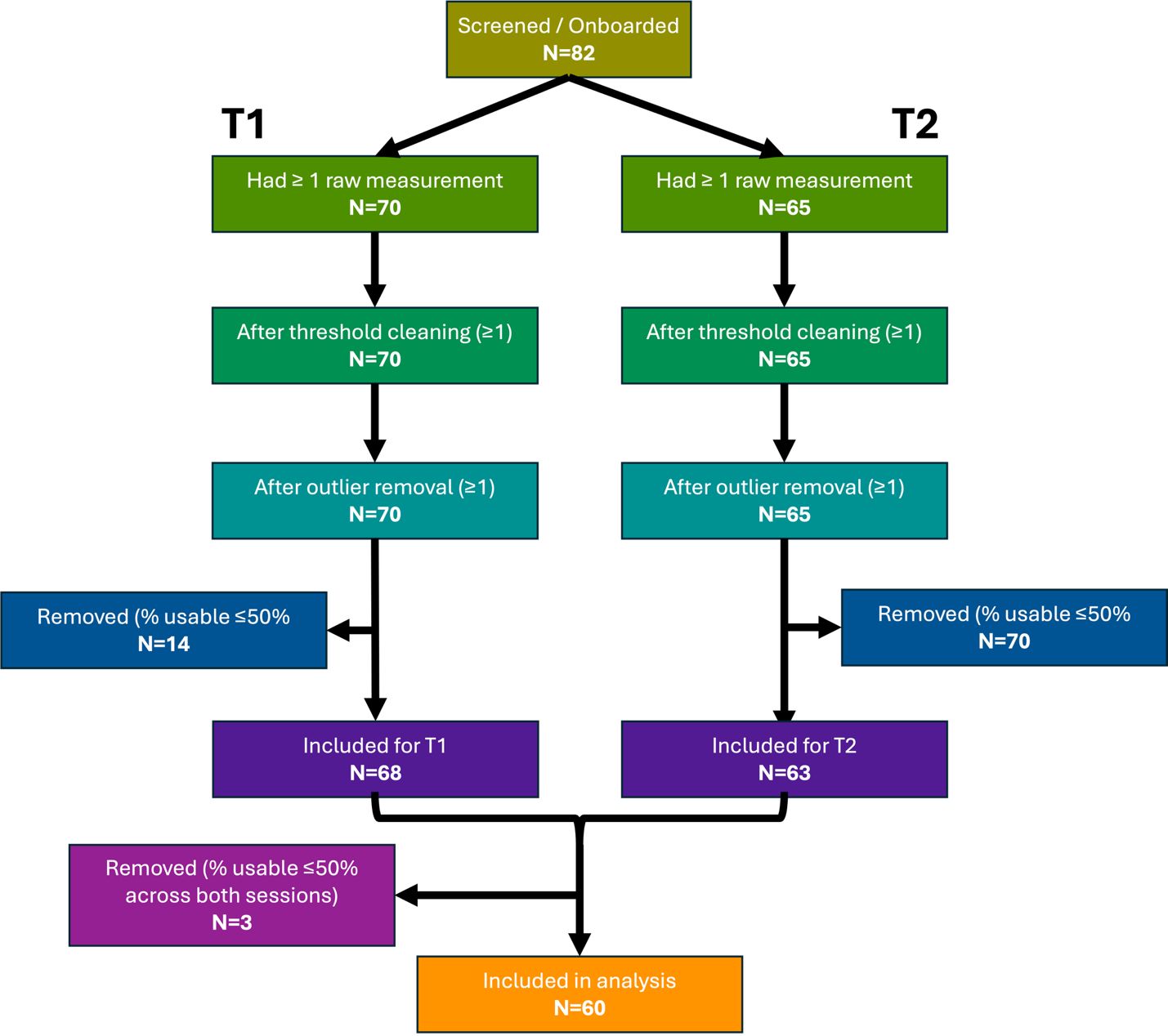


Figure S2. Flowchart of participant data availability and cleaning across timepoints. Boxes show N participants at each stage: screened, had ≥1 raw measurement, after data cleaning threshold values that failed when downloading data from the PART app (e.g., data labeled NA once downloaded from the app), after outlier removal (values >3 SD from session mean), removal due to <50% usable data per session, and included. Three participants were removed for having 50% when combining data from both sessions. Numbers are computed from the dataset and reflect all cleaning steps applied in the analysis pipeline.

|  |  | **Tolerability** | | |  | **Comfortability** | |  |
| --- | --- | --- | --- | --- | --- | --- | --- | --- |
| **Task** | **Yes/No** | **T1 (%)** | **Yes/No** | **T2 (%)** | **Yes/No** | **T1 (%)** | **Yes/No** | **T2 (%)** |
| PT | 33/35 | 94.3 | 28/29 | 96.6 | 30/35 | 85.7 | 26/29 | 89.7 |
| CLL | 28/34 | 82.4 | 20/27 | 92.3 | 25/34 | 73.5 | 22/26 | 84.6 |
| FM**_Dichotic_** | 32/34 | 94.1 | 27/2 | 100 | 28/34 | 82.4 | 25/27 | 92.6 |
| FM**_Diotic_** | 34/36 | 94.4 | 30/30 | 100 | 32/36 | 88.9 | 28/30 | 93.3 |
| STM | 36/37 | 97.3 | 29/30 | 96.7 | 35/37 | 94.6 | 29/30 | 96.7 |
| DSI | 37/38 | 97.4 | 30/30 | 100 | 34/38 | 89.5 | 28/30 | 93.3 |
| DIN | NA | NA | 7/7 | 100 | NA | NA | 7/7 | 100 |
| SRM | 29/35 | 82.9 | 20/27 | 74.1 | 25/35 | 71.4 | 19/27 | 70.4 |

Table S1. Self-reported tolerability and comfortability of participants. Participants were asked two yes or no questions, “Did you find the past task tolerable?” and “Were you comfortable during the last task?”. Percentages represent the total individuals who rated the task either comfortable or tolerable. PT = Pure tone thresholding task; CLL = Comfortable Listening Levels; FM = Frequency modulation. STM = Spectrotemporal modulation; DSI = Dichotic Sentence Identification; DIN = Digits in Noise; SRM = Spatial Release from Masking. Missing comfort data can be assumed missing at random, as no large systematic differences were found, barring the digits-in-noise task, where an error with the Qualtrics survey used to record participants’ responses, malfunctioned. However, we caution that some task-specific administrative errors may produce non-random patterns and future work should reduce administrative omissions and consider multiple-imputation where appropriate.

| **Task** | Paired t-test  *p*_adjusted_ |  | Rho  [95%CI] | Rho  *p*_adjusted_ |  |
| --- | --- | --- | --- | --- | --- |
| HHIE-S (Total) | 0.048 | * | 0.9 [0.83, 0.94] | 0 | * |
| CLL_M_ (dB SPL) | 0.733 |  | 0.3 [0.05, 0.52] | 0.023 | * |
| CLL**_C_** (dB SPL) | 0.733 |  | 0.21 [-0.05, 0.44] | 0.119 |  |
| CLL**_U_** (dB SPL) | 0.733 |  | 0.23 [-0.03, 0.46] | 0.097 |  |
| CLL**_CU_** (dB SPL) | 0.733 |  | 0.15 [-0.11, 0.39] | 0.253 |  |
| PT_Left 1kHz_ (dB HL) | 0.024 | * | 0.62 [0.43, 0.76] | 0 | * |
| PT_Right 1kHz_ (dB HL) | 0.037 | * | 0.45 [0.21, 0.64] | 0.002 | * |
| PTdc _0.5 kHz_ (dB HL) | 0.229 |  | 0.35 [0.11, 0.56] | 0.009 | * |
| PTdc _2 kHz_ (dB HL) | 0.229 |  | 0.69 [0.52, 0.8] | 0 | * |
| PTdc _4 kHz_ (dB HL) | 0.238 |  | 0.6 [0.41, 0.74] | 0 | * |
| FM**_DC_** (log_2_Hz) | 0.096 |  | 0.55 [0.34, 0.71] | 0 | * |
| FM**_DT_** (log_2_Hz) | 0.765 |  | 0.58 [0.37, 0.73] | 0 | * |
| STM (dB SPL) | 0.281 |  | 0.54 [0.32, 0.7] | 0 | * |
| SRT (dB SPL) | 0.096 |  | 0.54 [0.33, 0.7] | 0 | * |
| SRM**_C_** (dB TMR) | 0.857 |  | 0.36 [0.12, 0.57] | 0.008 | * |
| SRM**_S_** (dB TMR) | 0.096 |  | 0.57 [0.37, 0.72] | 0 | * |
| SRM (dB TMR) | 0.096 |  | 0.5 [0.28, 0.67] | 0 | * |
| DSI (Accuracy) | 0.428 |  | 0.68 [0.51, 0.8] | 0 | * |
| DIN (dB TMR) | 0.733 |  | 0.57 [0.36, 0.73] | 0 | * |

Table S2. False-discovery rate (FDR) corrections for paired sample t-tests and Pearson correlations. After Benjamini–Hochberg correction, paired t-tests revealed statistically significant mean changes for HHIE-S total score (q = 0.048) and for pure-tone thresholds at 1 kHz (left: q = 0.024; right: q = 0.037). Most tasks demonstrated significant test–retest correlations after correcting for FDR except for Comfortable listening levels (Comfortable, Uncomfortable, Confirm Uncomfortable). HHIE-S: Hearing Handicap Inventory for Elderly-Screening. CLL: Comfortable Listening Levels task; M: Masked; C: Comfortable; U: Uncomfortable; CU: Confirm uncomfortable. PT: Pure-tone threshold; DIN: Digits in noise. DSI: Dichotic Sentence Identification. SRT: Speech reception threshold. SRM: Spatial release from masking; C: Co-located; S: Separated. STM: Spectrotemporal modulation. FM: Frequency modulation; DC: Dichotic; DT: Diotic. * significance at an alpha level of 0.05.


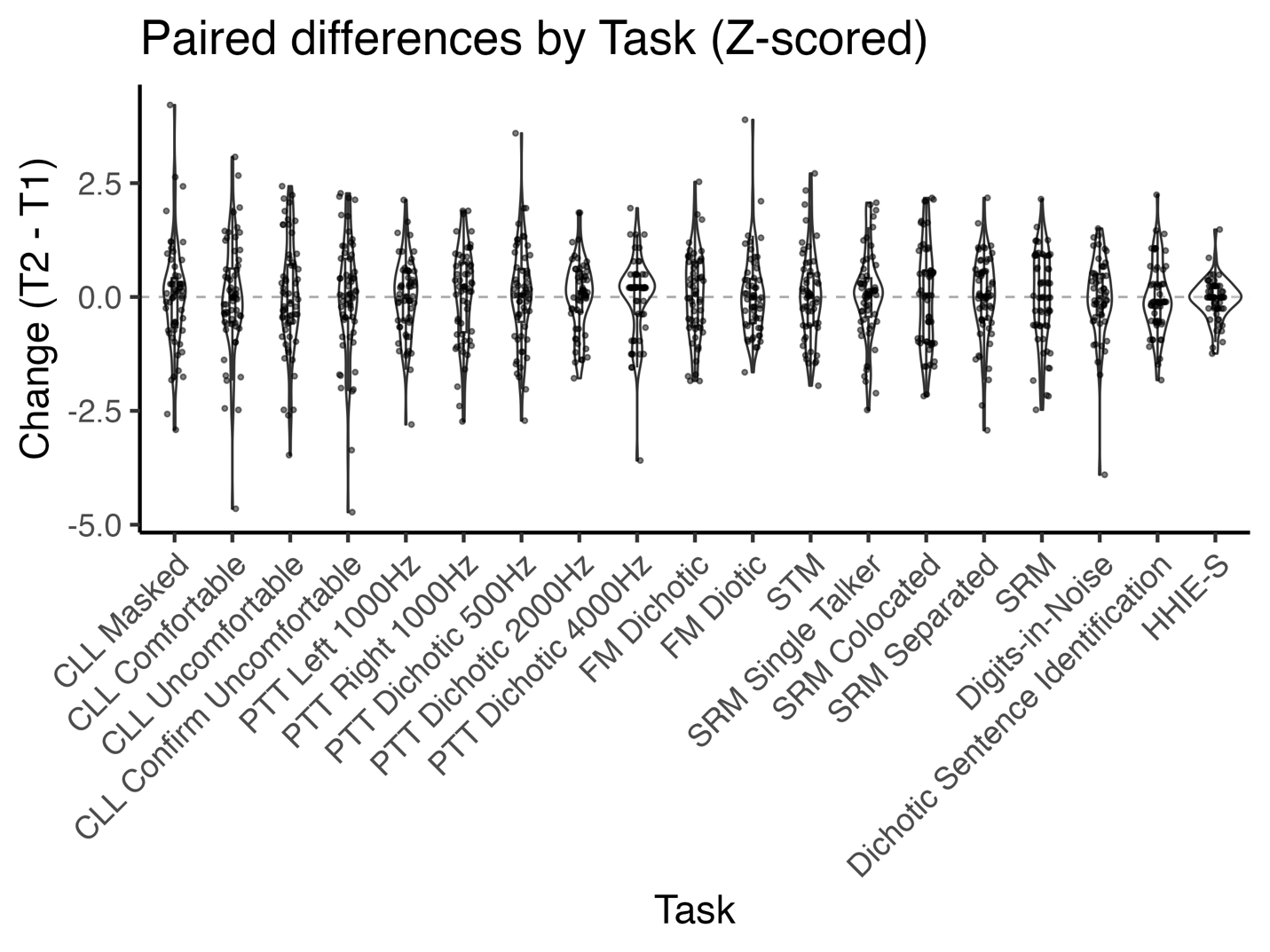
Figure S3. Z-scored change scores across timepoints. CLL: Comfortable Listening Levels; PTT: Pure Tone Thresholds; FM: Frequency Modulation; STM: Spectrotemporal Modulation; SRM: Spatial Release from Masking; HHIE-S: Hearing Handicap Inventory for Elderly-Screening.
